# Emerging therapeutic strategies for unmet need in neovascular age-related macular degeneration

**DOI:** 10.1186/s12967-023-03937-7

**Published:** 2023-02-21

**Authors:** Levon M. Khachigian, Gerald Liew, Kelvin Y. C. Teo, Tien Y. Wong, Paul Mitchell

**Affiliations:** 1 Vascular Biology and Translational Research, Faculty of Medicine and Health, School of Medical Sciences, University of New South Wales, Sydney, NSW 2052 Australia; 2 Centre for Vision Research, Westmead Institute for Medical Research, University of Sydney, Westmead, Australia; 3 Singapore National Eye Centre and Singapore Eye Research Institute, Singapore, Singapore; 4 Duke-NUS Graduate Medical School, National University of Singapore, Singapore, Singapore; 5 Tsinghua Medicine, Tsinghua University, Beijing, China

**Keywords:** Neovascular age-related macular degeneration, Vascular endothelial growth factor, VEGF receptors, Anti-VEGF therapy, Aflibercept, Ranibizumab, Bevacizumab, Brolucizumab, Faricimab

## Abstract

Neovascular age-related macular degeneration (nAMD) is a major cause of visual impairment and blindness. Anti-vascular endothelial growth factor (VEGF) agents, such as ranibizumab, bevacizumab, aflibercept, brolucizumab and faricimab have revolutionized the clinical management of nAMD. However, there remains an unmet clinical need for new and improved therapies for nAMD, since many patients do not respond optimally, may lose response over time or exhibit sub-optimal durability, impacting on real world effectiveness. Evidence is emerging that targeting VEGF-A alone, as most agents have done until recently, may be insufficient and agents that target multiple pathways (e.g., aflibercept, faricimab and others in development) may be more efficacious. This article reviews issues and limitations that have arisen from the use of existing anti-VEGF agents, and argues that the future may lie in multi-targeted therapies including alternative agents and modalities that target both the VEGF ligand/receptor system as well as other pathways.

Age-related macular degeneration (AMD) is the main cause of severe visual impairment and irreversible blindness in the industrialised world [1]. Late AMD, in the form of either neovascular (n) or atrophic AMD, is responsible for most vision loss. Although the prevalence of nAMD is lower than that of atrophy AMD, nonetheless it is responsible for most cases of severe vision loss [2]. Major advances in the treatment of nAMD over the past decade have occurred with the use of vascular endothelial growth factor (VEGF) inhibitors, most of which target VEGF-A. AMD has a prevalence of around 170 million, which is projected to increase to 288 million by 2040 [2]. Global prevalence in adults 45 years and over of any, early and late AMD is 8.7%, 8.0% and 0.4%, respectively, with early AMD being more common in people with European ancestry (11.2%) than Asians (6.8%) and no significant difference in the prevalence of late AMD [3]. AMD of any type is less frequent in people with African ancestry [3]. Approximately 1 in 10 persons with any AMD signs have nAMD [4]. In the US, about 200,000 new cases of nAMD are diagnosed each year [5].

## Pathophysiology and treatment concepts

**Fig. 1:**
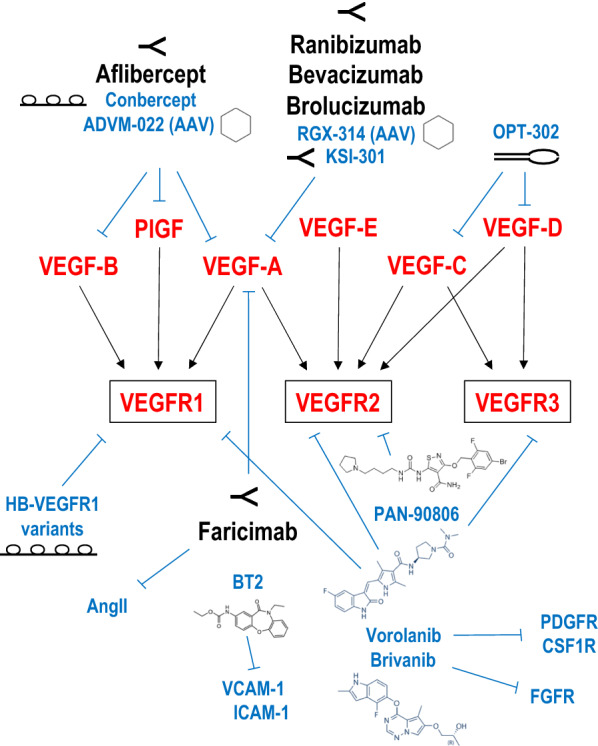
VEGF ligands and receptors. The VEGF ligand family (comprising VEGF-A, VEGF-B, VEGF-C, VEGF-D, virally encoded VEGF-E and placental growth factor (PlGF)) interacts selectively with 3 cell surface receptors (VEGFR1, VEGFR2, VEGFR3). Currently approved therapies are indicated in black font. Examples of emerging or experimental therapies are shown in blue font. Refer to text for detail

The pathogenesis of nAMD involves aberrent angiogenesis and macular neovascularization (MNV, also known as choroidal neovascularization (CNV)), vascular leakage, haemorrhage and scarring, which can lead to permanent vision loss [6–8]**.** A range of mediators have been implicated in this complex process including kinases [9], cytokines [10] and growth factors [11]; the most prominent is VEGF and its receptors (VEGFRs) [12] (Fig. 1). Most therapeutic attention on AMD and diabetic retinopathy (DR) has focused on VEGF-A and its receptors because of its dominant capacity to promote angiogenesis and vascular permeability, and its receptors [12].

AMD is classified clinically as early, intermediate or late. Early AMD is defined as the presence of medium-sized drusen (63–125 µm) without typical retinal pigmentary changes (hyper- or hypo-pigmentation) at the macula [13]. Intermediate AMD is defined as the presence of at least one large druse (> 125 µm) or extensive medium drusen and typical pigmentary changes. Late AMD refers to the presence of either nAMD (also termed “wet”) or atrophic AMD (also termed geographic atrophy). Early and intermediate AMD stages are often asymptomatic. There may be mild central distortion, particularly for near vision with low luminance. Late AMD is frequently symptomatic and reduces central vision for near and distance tasks. Neovascular AMD can progress rapidly over weeks to months, while atrophic AMD progresses more slowly over years to decades. Many patients report that the earliest symptoms of late AMD are distorted central vision, a dark patch (scotoma), which may be measured as a subtle visual field defect on microperimetry, and difficulty recognising faces [14]. Over 60% of AMD patients will develop the same stage of disease in both eyes [15]. In asymmetrically affected patients, the second eye then becomes affected in 19–28% of cases within 5 years of initial diagnosis [16].

The underlying nAMD lesion is the MNV (also known as CNV) complex which can be classified in terms of location—type 1 is restricted to the sub-retinal pigment epithelium (RPE) space; type 2 grows through the RPE and into the sub-retinal space; and type 3 is believed to originate within the retina [8]. A mixture of type 1 and 2 where neovascularisation in both the sub-retinal and sub-RPE spaces can also occur. In addition, a particular subtype known as polypoidal choroidal vasculopathy (PCV) [17, 18], similar to a type 1 CNV with dilated vascular elements has also been described as part of the spectrum of nAMD [8] and may be more prevalent among Asians [17, 18]. CNV is associated with other signs: retinal haemorrhage, intra- or sub-retinal fluid, pigment epithelial detachment (PED), exudate and subretinal fibrosis. AMD is diagnosed through a combination of retinal assessment through dilated pupils, and multimodal imaging, using colour fundus photography, OCT imaging, fundus fluorescein angiography (FFA), fundus autofluorescence (FAF) and more recently, OCT-angiography (OCTa) [14].

Atrophic AMD refers to the presence of geographic atrophy (GA) lesions that may be unifocal or multifocal [19]. Average progression rates of GA lesions is ~ 2 mm^2^/year with considerable variation [20]. Multimodal imaging is also used to diagnose and monitor GA progression. Reticular pseudodrusen (RPD), also known as subretinal drusenoid deposits, may be associated with more rapid progression of GA, and to a lesser extent, nAMD [21, 22].

AMD risk is influenced by genetic and non-genetic factors. Discovery of AMD associated genetic loci was the first major success of genome wide association study (GWAS) approaches [23]. Large AMD GWAS have since discovered 52 common and rare variants at 34 genetic loci independently associated with late AMD [24–26]. Association of very rare coding variants (frequency < 0.1%) in complement factor H (CFH), complement factor I (CFI) and tissue inhibitor of metalloproteinases-3 (TIMP3) suggest causal roles for these genes in AMD pathogenesis [24]. The alternative complement pathway (CFH, CFI, C2/complement factor B (CFB), C3) is primarily implicated in AMD risk [27] followed by the age-related maculopathy susceptibility2 (ARMS2) locus for which the gene product has not yet been identified [28]. TIMP3 codes for a matrix metalloproteinase inhibitor that regulates degradation of extracellular matrix, and is also implicated in Sorsby fundus dystrophy, a retinal degenerative disorder similar to AMD [29]. These 52 genetic variants together explain 27% of disease variability, which is over half the genomic heritability of AMD [24].

Smoking is the strongest modifiable risk factor for AMD and is associated with a doubling of late AMD risk [30, 31]. Current smoking is also associated with 10-year earlier age at onset of late AMD [30, 31]. Current smoking [32], and polymorphisms in *CFH* [33] and *ARMS2* [34] together account for up to 45% of risk for developing AMD [35]. Higher body mass index, an indicator of obesity, is also consistently associated with increased risk and earlier onset of developing AMD [36–38].

Dietary factors, especially intake of antioxidants, is also consistently associated with risk of developing AMD. Population studies such as the Blue Mountains Eye Study and others have shown a high dietary intake of lutein, zeaxanthin (carotenoids found in leafy green vegetables) and fish are associated with reduced risk of developing AMD [39–41]. Overall, Mediterranean and Oriental dietary patterns appeared to be protective against developing AMD, when compared to Western diets high in animal fats and red/processed meat [42]. The Age-Related Eye Disease Studies I and II were landmark clinical trials that confirmed nutrient supplements containing high doses of zinc and antioxidants (vitamin C, vitamin E, carotenoids, copper) can slow AMD progression in some people [43, 44].

Other factors with less consistent associations with AMD risk include: iris colour and sun exposure [30], alcohol intake [45], inflammatory markers such as C-reactive protein and white cell count [46, 47]. Cardiovascular risk factors, such as hypertension and dyslipidaemia are inconsistently linked with AMD risk [48], with elevated serum lipids associated with increased risk of intermediate AMD in some studies [49] but not others [50]. Long term aspirin use may be linked with a small increased risk of late AMD in a few studies [51, 52] but this has not been confirmed [53–55]. The risk of cataract surgery worsening early AMD is controversial. The latest Cochrane review to study the issue [56] found insufficient evidence to support cataract surgery as a risk factor for late AMD. Risk scores that include age, gender, smoking and drusen type perform well at discriminating persons who go on to develop AMD, with area under the receiver-operating curve of 0.85–0.91 [57–59].

## Current management

The present management of late AMD is focused on treating nAMD, as there are no proven treatments to date for atrophic AMD/GA.

### Emerging approaches to treating atrophic AMD (GA)

Recently pegcetacoplan, a C3 inhibitor delivered intravitreally, has been shown in two phase 3 trials (DERBY and OAKS) to significantly reduce the rate of grown of atrophic AMD/GA lesions when delivered monthly or every other month. [60–62]. This represents the first potential treatment to delay growth of GA, although visual acuity, reading vision and other functional parameters remained similar in treatment and sham groups. Approval from the US Food and Drug Administration (FDA) is pending and likely to occur in the near future. A number of other agents to treat GA are currently in late phase trials [60, 63–65].

GA is typically largely asymptomatic until the fovea is involved. Visual acuity does not correlate well with GA severity as the fovea may be spared despite extensive GA elsewhere [66], which may account for the lack of efficacy of pegcetacoplan on visual acuity. Clinical trials of GA using traditional visual acuity as an endpoint would need to be prohibitively long to detect differences due to slow growth of GA lesions. Alternative clinical endpoints are being explored, such as reading indices [67], and either single morphologic endpoints (enlargement of fundus autofluorescence (FAF) defects) or composite morphologic endpoints based on multimodal imaging, that may improve power to detect efficacy of interventions [68]. Complement inhibition is the most studied potential therapeutic intervention [69]. Drugs other than pegcetacoplan, such as eculizumab [70], lampalizumab [71] and tandospirone [72] have been investigated in clinical trials that have so far yielded disappointing results. Management of atrophic AMD/GA is not the focus of the present review.

### Current anti-VEGF therapies for nAMD

Intravitreal (IVT) anti-VEGF therapy is the standard of care for the treatment of nAMD (Table 1). Therapies include aflibercept [73], ranibizumab [74, 75], bevacizumab [76] which is currently used as off-label therapy but due to be registered, and brolucizumab [77]. Landmark registration trials for VEGF inhibitors demonstrate excellent visual outcomes when treated with monthly ranibizumab. The MARINA [78] and ANCHOR [79] trials showed gains of about 2 lines over 24 months. Similarly, VIEW [80] demonstrated non-inferiority of 2 monthly aflibercept compared to monthly ranibizumab. The Comparison of Age-Related Macular Degeneration Treatment Trials (CATT) found that bevacizumab had similar effectiveness to ranibizumab in treating nAMD [81], while the HAWK and HARRIER trials showed both 2 monthly and 3 monthly brolucizumab was non-inferior to 2 monthly aflibercept in treating nAMD [77]. Comparison between aflibercept, ranibizumab and bevacizumab for retinal disease suggest that aflibercept may have a slight benefit over ranibizumab and bevacizumab [82]. Ongoing CNV activity is associated with poorer visual outcomes [83, 84].

**Table 1:** Current anti-VEGF therapies for nAMD

Currently used therapies	Company	Type of therapeutic	Delivery route	FDA approval for nAMD
Ranibizumab/Lucentis	Lucentis, Genentech/Novartis	Antibody	IVT	2006
Aflibercept/Eylea	Regeneron/Bayer	Fusion protein “trap”	IVT	2019
Brolucizumab/Beovu	Novartis	Antibody	IVT	2019
Port delivery systems*	Genentech	Antibody	Implant	2021
Faricimab/ Vabysmo	Roche/Genetech	Bi-specific antibody	IVT	2022
Bevacizumab/Avastin	Roche/Genetech	Antibody	IVT	Used off label

IVT denotes intravitreal

^*^An already approved anti-VEGF agent (ie ranibizumab) not the port itself

Faricimab (Genentech/Roche) is a bispecific (dual targeted) antibody that simultaneously targets VEGF-A and angiopoietin-2 (Ang II), the latter being involved in distinct pathways that promote vascular permeability and inflammation [85]. In early 2022, two global Phase III studies (TENAYA (NCT03823287) and LUCERNE (NCT03823300) involving 1329 patients, reported that faricimab met its primary endpoint and showed potential to extend time between treatments up to 4 months for people with nAMD and was generally well tolerated [86]. Similar results were obtained in patients with diabetic macular edema (DME) in the YOSEMITE (NCT03622580) and RHINE (NCT03622593) Phase III trials involving 1891 individuals [87]. This follows the Phase II STAIRWAY trial (NCT03038880) which showed that monthly IVT faricimab was not superior to monthly ranibizumab [88], and Phase II AVENUE trial (NCT02484690) which showed that IVT faricimab given every 12 or 16 weeks was not clinically inferior to monthly ranibizumab for the treatment of nAMD [89]. With injections every 4 months rather than each month, faricimab could substantially reduce treatment burden and costs for patients and health care providers. Faricimab was approved by the FDA in January 2022 for the treatment of nAMD and DME.

New therapies with longer durability and better efficacy in early trials that target VEGF-A alone have not been as successful as the initial generation of anti-VEGF agents, largely due to unanticipated side effects. Brolucizumab has been associated with intraocular inflammation (~ 4%) [90, 91], retinal vasculitis and retinal artery occlusion [77]. Intraocular inflammation also occurs with abicipar pegol [92], a designed ankyrin repeat proteins (DARPin)-based drug that binds VEGF-A, exceeding 15% in Phase III trials (CEDAR and SEQUOIA, NCT02462928 and NCT02462486, respectively) as compared with less than 1% with ranibizumab [92–94]. Abicipar was rejected by FDA in June 2020 over risk/benefit concerns [94].

### Treatment regimens

Clinical trial treatment dosing regimens are often not reflective of real-world practice and high frequency of therapeutic interventions in registration trial design can result in a high treatment burden. In most clinical practice, non-monthly regimens such as the *pro re nata* (PRN) approach and the treat and extend (T&E) regimen have gained popularity with many favoring the later. The general principle behind non-monthly regimens is assessment of disease activity to determine the next management step taking into account personalised response to therapy. Briefly, in a PRN approach, disease status is assessed monthly, and treatment administered if disease is deemed active. In the T&E approach, treatment is administered at every visit, but treatment intervals are varied according to disease status [95]. These non-monthly regimens can reduce treatment burden to once every 3 to 4 months in some patients, while maintaining favourable visual outcomes [96, 97]. T&E is now the dominant treatment regime worldwide and future research directions are focused on further extending the speed and extent of increased intervals between injections. This points to the need for more durable agents and the different strategies taken to achieve this.

### Pharmacogenomics and personalised medicine

There is emerging evidence that a patient’s underlying genetic predisposition may affect response to existing anti-VEGF therapies. Lower risk genotypes of the *VEGFA, CFH, ARMS2* and *HTRA1* genes may be associated with better visual outcomes and potentially fewer injections [98–101]. Some of these genotypes were associated with poor response to one, but better response to a different anti-VEGF agent [102]. The effect of high risk alleles on anatomical and visual outcomes has been reported to be detectable even after long term treatment of up to 10 years [103]. The results raise the possibility that multi-targeted therapy could be personalised to individuals based on genetic risk scores, which could guide choice of therapy. Nonetheless, such pharmacogenetic scenarios remain in their infancy as a number of other studies have found very limited (to rare variants) [104] to no effect of high risk alleles on response to therapy [105, 106].

## Unmet need from use of current anti-VEGF therapies impacting quality of life

While anti-VEGF therapy has proven to be efficacious, several shortcomings highlight unmet need of this approach, thereby impacting the quality of life of patients suffering from and receiving treatment for nAMD. Table 2 summarises patient, therapeutic and healthcare system factors where there is unmet meet with current anti-VEGF therapies for nAMD. These factors are discussed further below.

**Table 2:** Unmet need

Factors	Unresolved issues	Comments
Patient factors	Non-adherence and/or non-compliance	Patient education and better understanding of disease and therapy
	Cost of therapy/visits	Individual healthcare jurisdiction cost and reimbursement policies
	High individual treatment burden	Need for more durable agents
	Poor prognosis	Need for more efficacious agents or regenerative therapies to reverse damage by nAMD
Therapeutic factors	Relatively short duration of action resulting in repeated treatments	Need for more durable agents
	Poor efficacy resulting in persistently active disease	Need for more efficacious agents
	Safety profile	Need for better preclinical safety models that can provide early safety signals before entry into clinical practice
Healthcare system factors	High treatment burden to society	Combination of more durable and efficacious agents may help address this unmet need by preventing/reversing blindness from nAMD
	Reimbursement and subsidies	Individual healthcare jurisdiction cost and reimbursement policies
	Increasing patient load	Need for more durable agents and/or more precise therapies to minimise unnecessary monitoring visits

### Suboptimal response or the response is not sustained

Despite favorable outcomes in most patients, 25–35% of nAMD patients either fail to respond optimally to current anti-VEGF therapies, exhibit late failure to therapy, or require intensive, frequent IVT treatment [107, 108]. Of the 35% who fail to respond optimally to therapy, over 10% worsen despite treatment, while another 25% show no improvement [83, 84]. Despite maximal intensive anti-VEGF therapy, over 60% of eyes have persistently active disease, which can result in poor long-term outcomes [84, 109]. Around 1 in 5 patients become “injection-intensive” needing treatment at least every 4 to 6 weeks [1]. This rigorous schedule can lead to high non-adherence/dropout over time, which further exacerbates the condition compromising the efficacy of anti-VEGF therapy [110–112]. This may be why poorer visual acuity outcomes are achieved in real world settings than in clinical trials [113–115].

In patients that achieve disease quiescence, suspending therapy may be detrimental and treatment often needs to be continued at regular intervals to maintain vision. Nguyen et al*.* reported a recurrence rate of > 40% in nAMD patients after treatment cessation following a 12-week interval [116]. Recent studies indicate that pre-treatment VEGF levels in the aqueous humor of nAMD patients correlated significantly with the likelihood of disease recurrence [117]. Moreover, in some nAMD patients with aggressive disease, continuation of anti-VEGF therapy even after achieving stability does not prevent disease recurrence [118–120]. The impact of suboptimal response and poor sustainability with resultant poor vision has significant impacts on patient reported outcomes. Patients were found to be willing to tolerate other inconveniences of receiving repeated anti-VEGF treatments if resultant vision was better [121–123]. CATT showed that 1 in 4 patients who needed aggressive anti-VEGF therapy developed some subretinal fibrosis within 2 years [124] and a greater risk of geographic atrophy in nAMD patients 2 to 5 years after start of therapy [125].

### Healthcare-related costs

Recurrent treatments for nAMD impose a high financial burden on health care systems (*e.g.* [126]*,*). Ross et al*.* examined compared the cost-effectiveness of aflibercept, bevacizumab and ranibizumab for treatment of DME and found that aflibercept and ranibizumab, respectively, were 31 and 20 times more expensive than bevacizumab [127]. Aflibercept and ranibizumab were not cost-effective relative to bevacizumab [128]. Off label use of bevacizumab for ophthalmic disease however, has helped mitigate cost for eye conditions in jurisdictions throughout Europe [129] and the US [130]. Anti-VEGF costs to patients are hugely variable in Asia; from comparative high cost in Indonesia with no government subsidies, to fully subsidized through public health insurance in South Korea and Japan [131–133]. In Australia, the regulatory framework disincentivizes use of bevacizumab for nAMD since the drug is unlisted and attracts no government subsidy [134]. There are also continuing issues regarding the safety of compounding bevacizumab for off-label intraocular use [135]. There is also increasing interest in biosimilars. Biosimilar medicines are close, but not identical versions of the original. For example, SB11 [136] and razumab [137] are ranibizumab biosimilars and may be available at lower cost. However, pharmacological issues of limited efficacy and durability persist with biosimilars. In addition to the cost effectiveness of therapy itself, there is significant burden of treatment to patients and care givers.

### Undesirable sequelae of anti-VEGF use

Existing anti-VEGFs are linked with some adverse events. These, while rare, can significantly impact vision. The major short term adverse effect of anti-VEGF injections is the risk of endophthalmitis, a serious outcome that occurs in approximately 1/3500 injections [138]. Early treatment with either IVT antibiotics, early vitrectomy, or both, is essential and can result in significant improvement in vision post-endophthalmitis [139]. Another major potential complication of anti-VEGF therapy is the risk of intraocular inflammation, which can also lead to irreversible vision loss if severe. The risk of this appears to be highest for the anti-VEGF agent brolucizumab, which is associated with a sixfold higher risk of intraocular inflammation compare to aflibercept [140]. Finally, a transient rise in intraocular pressure is often observed immediately post IVT injection of all anti-VEGF agents. This can sometimes be associated with “black outs” or sudden loss of vision as intraocular perfusion is compromised. In virtually all cases vision recovers over the next few minutes either spontaneously, or after anterior chamber paracentesis to relieve the intraocular pressure rise.

Long term use of anti-VEGF can be associated with adverse effects as well, though the causal relationship of long term anti-VEGF therapy and these adverse effects, as opposed to the natural history of nAMD, remains unclear and is subject of much investigation. Macular atrophy, an end stage phenotype of nAMD that can result in irreversible vision loss, has been reported to be associated with long term anti-VEGF use [141]. Ranibizumab treatment for nAMD over 2 years is associated with macular atrophy in about 30% of eyes [142] and longer term data has shown some detectable macular atrophy in 48% of eyes treated with anti-VEGF for 9 years [141]. Causality remains unclear and macular atrophy may represent the natural history of treated CNV [141–143]. Subretinal fibrosis is another end stage phenotype of nAMD associated with irreversible vision loss. Outcomes from CATT indicate that 1 in 4 patients on aggressive anti-VEGF therapy developed fibrosis within 24 months [124] with greater risk of geographic atrophy in nAMD patients 2 to 5 years after start of therapy [125] which again, could reflect the natural history of treated nAMD [141]. It should also be noted that untreated nAMD itself can result in subretinal fibrosis, which further confounds any potential relationship with long term anti-VEGF use.

There also remain some concerns about potential adverse effects stemming from systemic suppression of VEGF following long term anti-VEGF treatment [144]. While oral therapy in particular circumstances may be desirable, there is concern that systemic and chronic administration of agents that inhibit VEGF may lead to adverse events including kidney damage and hypertension [145] secondary to VEGF acting as a trophic factor in the retina and kidney. Though rare, repeated IVT injection of aflibercept and bevacizumab can result in serum drug levels rising above IC_50_ concentrations and reduced plasma free VEGF levels [146]. Serial injections of anti-VEGF in nAMD patients can elevate intraocular pressure which in some cases may require glaucoma therapy [147], although the incidence of this is low [148]. Long term IVT bevacizumab may increase risk of developing hypertension [149]. There is also some evidence that IVT anti-VEGFs may carry risk for systemic adverse thromboembolic events [150], however other studies have not found a link [151]. Further, systematic reviews have generally found low, if any, increased risk of thromboembolic events, which are acceptable when balanced against the clear efficacy in preventing vision loss [152].

There is evidence of cross regulation between VEGF ligands when a VEGF ligand is suppressed. Inhibition of one VEGF ligand by an approved anti-VEGF can induce expression of other VEGF ligands. Studies on mechanisms of tumour resistance indicate that resistance to aflibercept coincides with increased levels of VEGF-C [153], a lymphangiogenic growth factor [154] and that resistance to bevacizumab coincides with increased levels of PlGF and VEGF-D [155]. VEGF-C and VEGF-D expression is increased in human brain and tumour derived endothelial cells exposed to bevacizumab [156]. VEGF-D has been implicated in lymphangiogenesis, tumor growth and metastatic spread [157]. In nAMD patients, while IVT injection of bevacizumab reduces VEGF-A levels in the aqueous humor, this elevates levels of VEGF-C and a range of other pro-angiogenic and pro-inflammatory factors [158].

Overall, these shortcomings can result in unfavourable outcomes and potentially visually significant complications in some patients. Longer lasting, alternative agents that achieve the same or better efficacy with fewer injections, could help to alleviate many of these shortcomings.

## Emerging therapies

### *Mono-targeted (anti VEGF) therapies in development for nAMD*

Targeting the VEGF/R system has undoubtedly prevented blindness in millions of nAMD patients and improved quality of life and workforce productivity. However, given the shortcomings of current anti-VEGF therapy, there remains a need to identify other types of agents and modalities exploiting this pathway, several of which are summarized in Table 3 and depicted in Fig. 1.

**Table 3:** Emerging or attempted therapies for nAMD

Drug or modality	Company or Institution	Type of agent	Delivery route	Phase	Clinical trials.gov
Abicipar pegol	Allergan	DARPin	IVT	III, terminated	NCT02462928, NCT02462486
Conbercept/Lumitin	Chengdu Kang Hong Biotech	Fusion protein “trap”	IVT	III, terminated	NCT03577899, NCT03630952
PAN-90806	PanOptica	Small molecule	Eye drops	I/II	NCT03479372
RGX-314	RegenxBio	AAV8 gene therapy delivering anti-VEGF Fab	Subretinal	II	NCT04832724
ADVM-022	Adverum	AAV7m8 gene therapy delivering aflibercept	IVT	I	NCT03748784, NCT04645212
KSI-301	Kodiak	Antibody biopolymer conjugate	IVT	IIb/III	NCT04049266
OPT-302	Opthea	Fusion protein “trap”	IVT	III	NCT04757610, NCT04757636
EYP-1901	Eyepoint	Small molecule in Durasert	IVT	I	NCT04747197
Pluripotent stem-cells	London Project to Cure Blindness	hESC-derived RPE monolayer	Transplantation	I	NCT01691261
Pluripotent stem-cells	Highway Program for Realization of Regenerative Medicine	Autologous iPSC-derived RPE cell sheet	Transplantation	UMIN000011929	
Heparin-binding VEGFR1 variants	University of California San Diego	Fc fusion proteins	IVT	N/A	
OXU-005	Oxular	Small molecule in Oxuspheres	IVT	N/A	
Brivanib	Nantong University	Small molecule	IVT, gavage	N/A	
AGX51	Memorial Sloan Kettering Cancer Center	Small molecule	IVT	N/A	
THR-687	Oxurion	integrin receptor antagonist	IVT	N/A	
Vasotide	Beth Israel Deaconess Medical Center	peptidomimetic	Eye drops	N/A	
BT2	UNSW	Small molecule	IVT	N/A	
Sunitinib	Johns Hopkins University	Small molecule	IVT	N/A	
APX2009, APX2014	Indiana University	Small molecule	Systemic	N/A	

N/A denotes not (yet) available, IVT denotes intravitreal, iPSC denotes induced pluripotent stem cells, hESC denotes human embryonic stem cell, RPE denotes retinal pigment epithelium, AAV denotes adeno-associated virus, DARPins denote designed ankyrin repeat proteins

^*^an already approved anti-VEGF agent (ie ranibizumab) not the port itself

### Protein-based agents

Inspired by the need for longer acting VEGF inhibitors, and exploiting the heparin affinity of VEGFRs, Ferrara and colleagues recently developed heparin-binding variants of VEGFR1 that compare favourably with aflibercept in rodent models of retinal neovascularization [159]. Fc-containing proteins with the D3 (Ig-like) domain of VEGFR1 (*e.g.,* V1233, V233) bound to bovine vitreous in *vitro* and suppressed retinal angiogenesis following IVT injection and laser-induced CNV in mice. These Fc fusion proteins were detectable in serum after IVT delivery albeit at levels less than aflibercept [159].

Conbercept (Lumitin; Chengdu Kang Hong Biotech) is a fusion protein comprising the extracellular domain 2 of VEGFR1 and extracellular domains 3 and 4 of VEGFR2 combined with the Fc portion of human IgG_1_. Like aflibercept, conbercept is designed to bind VEGF-A, VEGF-B and PlGF [160]. Monthly IVT conbercept appears to improve visual acuity in nAMD patients with no serious adverse reactions or complications [161]. However potential concerns have been raised about the extent of CNV, prior patient treatment and unresolved macular edema secondary to insufficient macular deturgescence suggesting active disease requiring further treatment [162]. The PANDA-1 and PANDA-2 Phase III trials for nAMD involving 1157 participants were terminated in 2021 on the basis that the desired primary endpoint, notably conbercept non-inferiority compared with aflibercept was not met (NCT03577899 and NCT03630952).

OPT-302 is a novel “trap” molecule comprising Ig-like domains 1 to 3 of the extracellular domain of human VEGFR3 fused to the Fc fragment of human IgG_1_ that binds to VEGF-C and VEGF-D, blocking their activation of VEGFR2 and VEGFR3 [163]. In NCT02543229, Dugel et al*.* found that IVT OPT-302 was well tolerated with or without ranibizumab (0.5 mg) up to the highest dose of 2 mg given as 3 injections once every 4 weeks [163]. Although VEGF ligand levels were not measured in the aqueous humour, conceptually at least, combining OPT-302 with anti-VEGF-A therapies may prevent mechanistic escape following VEGF-A suppression. OPT-302 is recruiting for Phase III trials with and without either ranibizumab (ShORe trial, NCT04757610) or aflibercept (COAST trial, NCT04757636), each with 990 nAMD patients.

### Small molecule-based therapy

Small molecules offer a range of potential advantages over antibody based drugs such as lower costs of manufacture, longer shelf life and reduced need for cold chain transport, oral administration and drugability [164]. The tyrosine kinase inhibitor sunitinib, a small molecule commonly used to treat renal cell carcinoma, has recently been repurposed as an experimental therapy for nAMD [165]. Single IVT or subconjunctival injection of sunitinib, in a non-inflammatory biodegradable polymer-based microparticle formulation (polylactic-co-glycolic acid (PLGA) and PLGA conjugated to polyethylene glycol (PEG)), provided sustained suppression of choroidal neovascularisation in mice over several months. In separate experiments, mice given IVT VEGF into each eye following injection of sunitinib microparticles (as compared with microparticles alone) showed significant reduction in the number of adherent intravascular leukocytes, indicating sunitinib suppression of VEGF-induced leukostasis. Delivery IVT of sunitinib microparticles in rabbits caused self-aggregation which remained localised and efficacious over several months with no intraocular inflammation or apparent retinal toxicity [165].

Eye drops are being developed as potential monotherapy or to facilitate an increased interval duration between IVT injections of standard therapy or for use after IVT injections to further stabilise active disease. Drops would provide convenience, increasing adherence and compliance by patients and caregivers through fewer clinic visits and reduce risk of infection from IVT injections. PAN-90806 (PanOptica), a small molecule that binds VEGFR2 inhibiting its tyrosine kinase activity, is being developed as a once-daily eye drop suspension for nAMD (NCT03479372) [166]. An earlier trial with a different formulation showed punctate keratopathy due to off-target suppression of corneal epithelial EGFR [167].

### Gene therapy

Adverum is developing a gene therapeutic approach for nAMD using ADVM-022 in which a proprietary vector capsid (AAV.7m8) delivers an aflibercept coding sequence. ADVM-022, administered by IVT injection was granted fast track designation by FDA in late 2018. Thirty patients completing 2 years in the Phase I OPTIC trial **(**NCT03748784) are being enrolled into an extension trial (NCT04645212) which will run for up to 5 years. Gelfman et al*.* reported the efficacy of ADVM-022-derived aflibercept in a CNV model involving non-human primates. ADVM-022 given 13 months prior to laser-induced CNV, prevented CNV lesions to the same extent as aflibercept delivered at the time of lasering [168, 169] demonstrating that one IVT delivery of ADVM-022 is safe and could provide a potential long-term treatment option for nAMD. Ding et al*.* performed suprachoroidal injections of RGX-314, an adeno-associated virus serotype 8 (AAV8) vector expressing an anti-VEGF-A Fab in rats and suppressed VEGF-inducible vasodilation and vascular leakage [170], building on earlier studies also in rats injecting RGX-314 subretinally [171]. RegenxBio is conducting a Phase II study in 60 subjects with nAMD in which RGX-314 is delivered by subretinal administration (NCT04832724). While gene therapy provides an alternative means of therapeutic intervention, it brings risk of immunogenicity in relation to adenoviral vectors, and risk of transgene integration in relation to retroviral and lentiviral vectors, or inability to carry large transgenes by AAV vectors.

### Other antibody-based strategies

Kodiak is developing KSI-301, an anti-VEGF antibody biopolymer conjugate (ABC) platform comprising a humanized IgG_1_ antibody binding all isoforms of VEGF-A [172] with an inert immune effector function and a biopolymer designed to increase intraocular durability. In the Phase IIb/III DAZZLE trial (NCT04049266) involving 550 nAMD patients, KSI-301 will be administered IVT at 12, 16 and 20 week intervals and compared against aflibercept once every 4 weeks for 3 consecutive months, followed by once every 8 weeks. The Phase Ib study (NCT03790852) showed 66% of nAMD patients achieved a 6 month or longer treatment-free interval and 78% had a 4 month or longer interval after 12 months [173].

Port delivery systems (PDS), enabling surgically implantable reservoirs, have been developed to facilitate continuous delivery of anti-VEGF agents such as ranibizumab inside the eye by passive diffusion [174]. PDS could potentially be used to deliver other therapeutics through sustained release and refilled months later. The Phase III ARCHWAY trial (NCT03677934) in 418 subjects with fixed 24 week refills revealed that 98.4% of PDS patients went 6 months without intervention and achieved vision outcomes equivalent to patients receiving monthly IVT ranibizumab. However, there was a significantly higher rate of ocular adverse effects, particularly endophthalmitis and vitreous haemorrhage in the PDS arm compared to monthly ranibizumab arm [175][175] prompting further virtual reality training strategies on implantation to mitigate risk [177]. The Phase IIIb VELODROME trial (NCT04657289) will evaluate PDS and ranibizumab refill (100 mg/ml) delivered every 36 weeks as compared with every 24 weeks. FDA approved PDS with ranibizumab for the treatment of nAMD in October 2021.

### *Multi-targeted therapies in development for nAMD*

Despite the promise of reduced treatment burden in nAMD patients brought about by brolucizumab, the future may lie with multi-target interventions. This is because considerable research suggests that factors beyond VEGF, such as other growth factors, chemokines and cytokines, also mediate the pathogenesis of nAMD [12]. Angiogenesis and inflammation underpinning nAMD involves signalling and transcriptional regulation mediated by extracellular signal-regulated kinase-1/2, p-ERK) [9], monocyte chemoattractant protein-1 (MCP-1/CCL2) [178], intercellular adhesion molecule-1 (ICAM-1) [178], vascular cell adhesion molecule-1 (VCAM-1) [178], interleukin-1β (IL-1β) [179] and IL-6 [180]. This may account for the inadequacy of strategies solely targeting the VEGF system [181, 182] and points to the therapeutic potential for strategies that also target other mediators of nAMD. For example, faricimab targets two distinct pathways, VEGF-A and Ang-2. Lessons emerging from cancer therapy suggest that simultaneous blockade of multiple pathways can make it harder for tumours to bypass therapy [183]. Indeed, resistance that develops to kinase inhibitors at least in melanoma patients may arise from reactivation of signalling pathways (or activation of parallel pathways) or immune system modulation [184]. There is also major need for agents with greater efficacy (to improve response) and durability (to reduce frequency of injection) for nAMD that may be achieved through multi targeting. For example, while aflibercept, a soluble decoy VEGF receptor, inhibits VEGF-A and VEGF-B it also binds placental growth factor (PlGF), which may account for its prolonged efficacy compared to the mono-targeted anti-VEGF-A antibodies ranibizumab and bevacizumab [82–84]. Several such strategies in development (Table 3) and are described below. It is unclear at this stage as to whether multi-targeted therapies are prone to more unpredictable side effects in the long-term.

### Small molecule-based therapy

A range of small molecules have been tested as multi-target therapeutics in preclinical models of nAMD. For example, brivanib, a pyrrolotriazine-based dual receptor tyrosine kinase inhibitor of FGFR1/R2 and VEGFR1/R2/R3 [185, 186] delivered IVT or by oral gavage blocked reduced CNV leakage and area following laser-induced CNV in mice [187]. Wojnarowicz et al*.* identified a first-in-class small molecule, AGX51, from an in silico screen, that caused ubiquitin-mediated Id protein degradation, G_0_/G_1_ arrest and reduced endothelial cell viability. IVT administration of AGX51 reduced CNV following laser injury in mice, and the AGX51/aflibercept combination had greater efficacy than aflibercept alone [188]. Hu et al*.* demonstrated that HR-687, a pan RGD (arginylglycylaspartic acid) integrin receptor antagonist that blocks the principal RGD integrins α_v_β_3_, α_v_β_5_ and α_5_β_1_, inhibits VEGF-induced leakage in the mouse retina and retinal leakage in cynomolgus monkeys following laser-induced CNV as effectively as ranibizumab [189]. Additionally, Sidman et al. found that vasotide, a small cyclic retro-inverted peptidomimetic, D(Cys-Leu-Pro-Arg-Cys) which binds neuropilin-1 (NRP-1) and VEGFR1 can inhibit retinal CNV in a laser-induced African Green monkey model after eye drop delivery [190].

We recently identified BT2, a dibenzoxazepinone that can suppress not only VEGF-A, but also p-ERK, MCP-1, ICAM-1, VCAM-1, IL-1β and IL-6 among a range of other pro-angiogenic and pro-inflammatory mediators relevant to nAMD [191, 192]. This includes transcription factors (*e.g.,* Egr-1, c-Rel/NF-κB, KLF) and pro-angiogenic chemokines (*e.g.,* CXCL1, CXCL3, CXCL8, CCL20). BT2 inhibits endothelial cell proliferation, migration, tubule formation and angiogenesis in mice bearing Matrigel plugs [191]. IVT BT2 reduced retinal permeability in rats as effectively as aflibercept at the same dose but needed threefold fewer injections than aflibercept [191]. BT2 also reduced retinal vascular permeability in rabbits induced by VEGF-A [191]. BT2 suppressed laser injury-induced CD31, pERK, VEGF-A and FosB/AP-1 (a family of transcription factors that regulates VEGF-A [193, 194]) expression in the retina [191]. The catalytic oligonucleotide, Dz13, provides another example of a molecular approach that inhibits VEGF-A and retinal neovascularization by targeting transcription factor (c-Jun/AP-1) controlling its expression [195, 196]. Thus, strategies that suppress regulatory factors other than merely VEGF, could potentially assist patients resistant to standard anti-VEGF therapy, or may permit a longer duration of action, as suggested by faricimab.

### Ocular reservoirs

Biodegradable reservoirs implanted in the vitreous provide an alternative approach. Eyepoint Pharmaceuticals is developing EYP-1901, an indolinone-based small molecule tyrosine kinase inhibitor (vorolanib/CM082/X-82) [197] in Phase I trials for nAMD (DAVIO, NCT04747197). EYP-1901 blocks VEGFR1, R2 and R3 but also targets the platelet-derived growth factor receptor (PDGFR $\mathrm{\alpha }$ and ß) and colony stimulating factor 1 receptor (CSF1R), and is delivered IVT in a bioerodible (Durasert) platform for potential twice-yearly sustained delivery [198].

OXU-005 (Oxular) is developing an alternate sustained release strategy of a proprietary narrow-spectrum kinase inhibitor using a biodegradable polymer system (Oxuspheres) which seeks to provide up to 12 months’ treatment after single administration into the suprachoroidal space [199].

### Systemic delivery

Agents that demonstrate efficacy in the retina following systemic delivery have been developed. This could potentially avoid the potential damaging effects of IVT injection and improve patient non-compliance. This includes small molecule inhibitors of reduction–oxidation factor 1–apurinic/apyrimidinic endonuclease 1 (APE/REF-1). APE/REF-1 redox activity regulates retinal endothelial cell growth, migration and tubule formation [200]. Intraperitoneal administration of APX2009 and APX2014 (50 mg/kg, twice daily, 5 days on/2 days off), blocked REF-1 redox signaling and attenuated laser-induced CNV in mice [200]. The likely increased risk of side effects from systemic medication administration should be balanced against benefits of systemic administration.

### Stem cell-based therapy

Stem cell-based experimental therapies, while in their infancy, have been tested in AMD patients. IVT administration of adipose tissue–derived “stem cells” in those with non-neovascular AMD caused severe vision loss (NCT02024269) [201]. This was associated with a range of pathologic effects including hemorrhagic retinopathy, vitreous haemorrhage, ocular hypertension, retinal detachment or lens dislocation. Transplantation of an autologous induced pluripotent stem-cells (iPSC)-derived RPE cell sheet in a patient with nAMD did not improve or worsen BCVA after 1 year and while cystoid macular edema was present, did not cause serious adverse events after 25 months (UMIN000011929) [202]. In a Phase I study (NCT01691261), da Cruz and colleagues delivered a synthetic basement membrane-based patch made of RPE that had been differentiated from human embryonic stem cells into the subretinal space of 2 patients with neovascular AMD. Patch transplantation was achieved using biomicroscopy and optical coherence tomography. This resulted in visual acuity gain of 29 and 21 letters in each patient, respectively, over a year [203], suggesting the safety and feasibility of stem cell-based RPE regenerative therapy for AMD.

## Future directions and conclusions

Since their first use as IVT drugs with nAMD patients 15 years ago [79], anti-VEGF therapies have transformed the treatment of macular degeneration and largely replaced less-effective treatments, such as photodynamic therapy [204]. Anti-VEGF agents have reduced incident legal blindness and visual impairment caused by nAMD, decreased economic and societal costs [205] and improved vision-related quality of life [206]. However, there remains unmet clinical need for improved therapies for nAMD since many patients do not respond optimally, lose response over time, or exhibit sub-optimal durability. Many patients in real-life clinical settings receive fewer anti-VEGF injections than those in clinical trial settings, and this can result in poor visual outcomes. There is a need for longer acting agents to reduce injection frequency, treatment burden, and for agents that do not leak into the systemic circulation from the vitreous.

Expansion of targets beyond VEGF-A is a promising strategy to address the contribution of non-VEGF mediated pathways to the pathogenesis and clinical manifestation of nAMD. Other agents and modalities exploiting the VEGF system and alternate pathways include heparin-binding variants of VEGF receptor 1, conbercept and iPSC-derived cells. Ultimately, a combination of approaches targeting the VEGF system concurrently with other key processes may be needed to satisfy unmet need in the treatment of nAMD. This could also allow for personalisation of treatment. Heterogeneity in clinical response to current VEGF-based therapies in nAMD suggest that different pathways predominate between individual patients. Targeting multiple pathways could improve response, prevent resistance and underpin future tailored treatments for nAMD and other neovascular/exudative retinal disorders.

## Data Availability

Not applicable.
